# *Arcanobacterium pyogenes* and encrusted pyelitis

**DOI:** 10.2144/fsoa-2019-0021

**Published:** 2019-12-09

**Authors:** Albert Semaan, Georges Abi Tayeh, Josselin Abi Chebel, Rabih Hallit, Matta Matta, Pascal Hajj

**Affiliations:** 1 Urology Department, Faculty of Medicine, Saint-Joseph University, Beirut, Lebanon; 2 Faculty of Medicine, Holy Spirit University of Kaslik – USEK, Lebanon; 3 Infectious Disease Department, BelleVue Medical Center, Mansourieh, Lebanon; 4 Faculty of Medicine, Saint-Joseph University, Beirut, Lebanon; 5 Urology Department, BelleVue Medical Center, Mansourieh, Lebanon

**Keywords:** *Arcanobacterium pyogenes*, atypical pathogens, encrusted pyelitis, human, pyelitis, renal calcifications, *Trueperella pyogenes*, urinary tract infections

## Abstract

**Aim:**

*Trueperella pyogenes* is known to affect cattle, but was never isolated as a cause of human urinary tract infections.

**Clinical case:**

A 69-year-old male presented for recurring low urinary tract symptoms after a 20-day ciprofloxacin regimen for prostatitis. He previously underwent open right nephrolithotomy and left ureterovesical junction reimplantation for an iatrogenic distal ureteral stricture. Computed tomography showed spontaneous cortical calcifications; renoscopy was performed and deep cultures from the pelvis were taken; culture on chocolate agar revealed *T. pyogenes*. Intravenous teicoplanin for 3 weeks resulted in resolution of low urinary tract symptoms with regression of bladder and ureteral thickening.

**Conclusion:**

*T. pyogenes* can cause encrusted pyelitis in humans especially evoked in a context of persisting or recurring urinary tract infections.

## Lay abstract

We present a case report of a 69-year-old male presenting with persisting low urinary tract symptoms after a ciprofloxacin regimen received for prostatitis. Diagnostic work-up revealed encrusted pyelitis based on the demonstration of spontaneous renal cortical calcifications on imaging, and growth of the bacteria *Trueperella pyogenes* on chocolate agar. Treatment with intravenous teicoplanin given for 3 weeks resulted in permanent resolution of low urinary tract symptoms.

## Case presentation

We herein report the case of a 69-year-old male with a past medical history of hypertension, dyslipidemia and a remote history of smoking (stopped 15 years ago), who works as a car painter and has no direct contact with animals, consulting for recurring lower urinary tract symptoms (LUTS) after completing a 20-day course of ciprofloxacin for prostatitis. His past urologic history included urolithiasis for which he underwent right open nephrolithotomy, left ureterovesical junction reimplantation for most likely iatrogenic distal ureteral stricture, benign prostatic hyperplasia treated with tamsulosin and recurring macrohematuria with sterile urine cultures. He also had an episode of acute prostatitis 1-year prior, treated by a 20-day course of ciprofloxacin with persistence of LUTS and sterile pyuria. His current medication regimen consists of aspirin, atorvastatin, vitamin D, perindopril amlodipine and tamsulosin.

A written informed consent was obtained from the patient for publication of his case report and any accompanied images. A copy of the written consent is available for review. The patient signed the written informed consent in his treating physician’s clinic.

**Figure 1.**
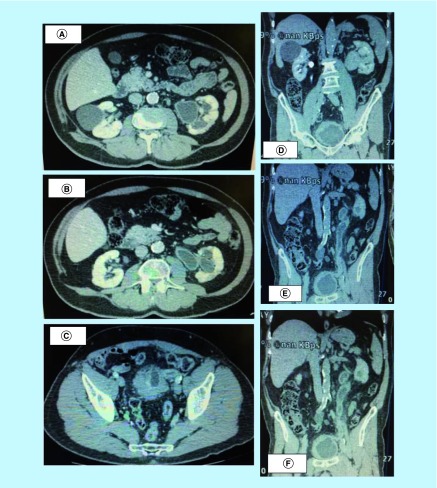
(A–C) Abdominopelvic computed tomography scan before treatment with teicoplanin showing calcification and urothelial wall thickening. **(D–F)** Abdominopelvic computed tomography scan before treatment with teicoplanin showing calcification and urothelial wall thickening.

Sperm culture showed no bacterial growth. Abdomen and pelvis computed tomography (CT) scan performed before injection of intravenous (IV) contrast showed a multiscarred left kidney with spontaneously hyperdense cortical images, likely in keeping with cortical calcifications, associated to multiple cortical cysts, some of them spontaneously hyperdense. A tomographic complement after IV contrast injection with acquisitions obtained at portal and delayed phases showed a multiscarred left kidney with a dilated tortuous hypotonic ureter and a thickened wall showing significant enhancement with surrounding fat streaking, multiple nonenhancing filling defects in the left renal pelvis (Figure 3), and satellite lymph nodes measuring up to 12 mm of lesser diameter. A thickened multidiverticular bladder wall, suggestive of bladder and upper tract urothelial tumor (Figure 1) was also noted. Urine pH was 6.5.

**Figure 2.**
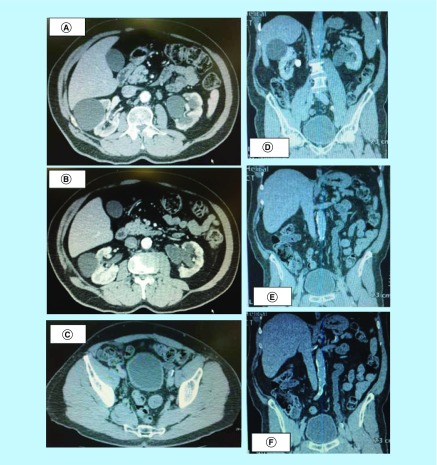
(A–C) Abdominopelvic computed tomography scan after treatment with teicoplanin showing complete regression of urothelial calcification and mucosal thickening. **(D–F)** Abdominopelvic computed tomography scan after treatment with teicoplanin showing complete regression of urothelial calcification and mucosal thickening.

**Figure 3.**
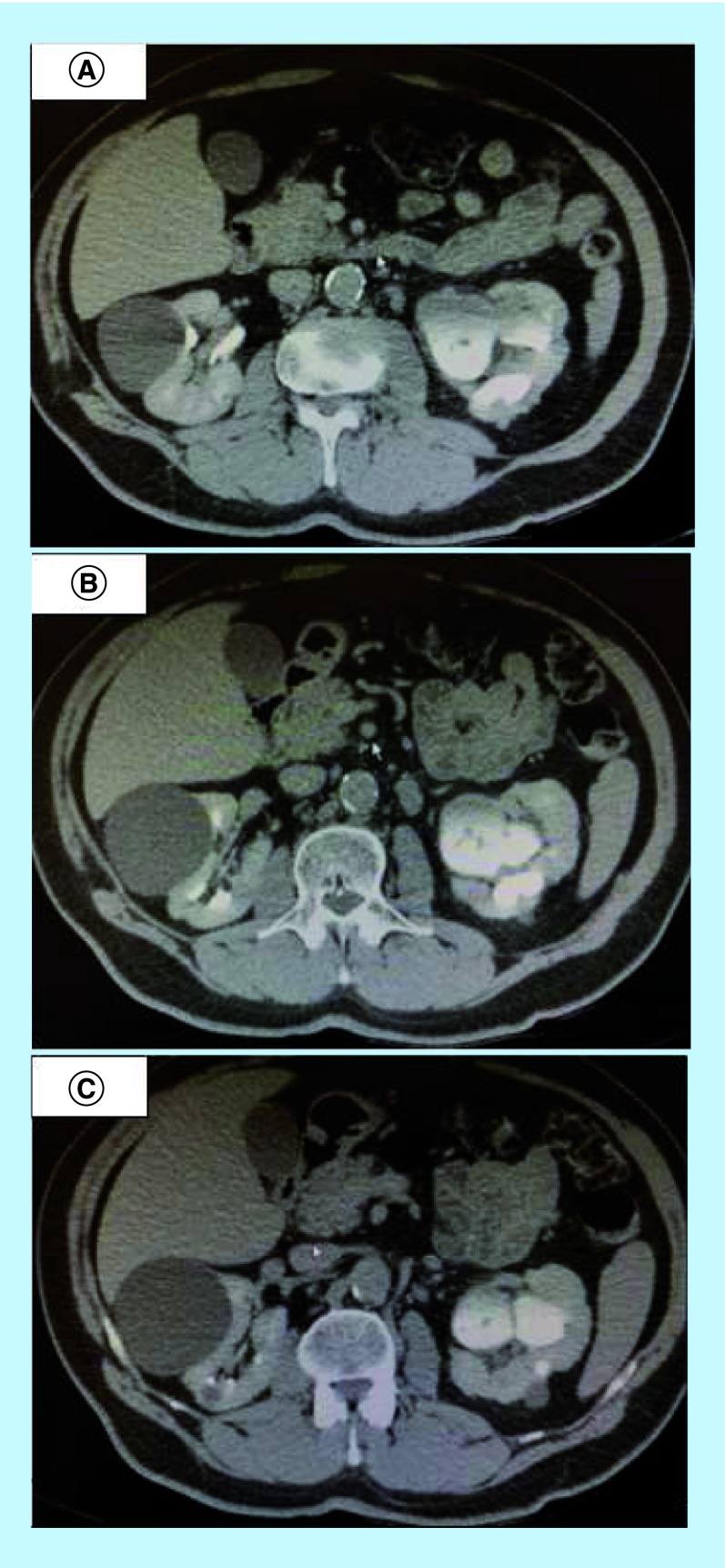
(A–C) Abdominal computed tomography scan showing multiple nonenhancing filling defects on the left renal pelvis at delayed phase before treatment with teicoplanin.

A cystoscopy with ureteroscopy and left ureteral and pelvic biopsies were performed showing a follicular cystitis, inflammatory ureteral mucosa, granulation tissue, ectatic lymph vessels, congestive and edematous changes, with no signs of malignancy. Renal pelvis showed no papillary tumor but cloudy, grayish urine with floating whitish debris instead. Those findings were suggestive of atypical infections. Deep cultures from the pelvis were taken showing no bacterial growth of usual pathogens. Therefore, work-up for atypical infections was initiated. PCR for tuberculosis was negative but specific culture on media for corynebaterial infections, chocolate agar *per se*, showed a growth of *Trueperella pyogenes*, formerly known as *Arcanobacterium pyogenes*. Pathogen identification was done by biochemical testing through an analytical profile index. Trypticase soy agar/broth with defibrinated sheep blood was the special media used for culture. The patient was treated with IV teicoplanin for 3 weeks. Antibiotic regimen included a bolus loading dose then a maintenance perfusion dose given over 30 min. Antibiotic treatment resulted in resolution of his urinary symptoms. Teicoplanin was used based on susceptibility tests performed *in vitro* on the isolated strain. Teicoplanin, along with vancomycin, has been proven to be the antibiotic to which all isolates of corynebacterial species were susceptible [1]. This is the main reason behind the choice of teicoplanin for management of the patient.

Patient management was performed on regular floor by a multidisciplinary team composed of urology and infectious diseases specialists. The treatment strategy was chosen based on clinical criteria since the patient demonstrated hemodynamic stability since his initial presentation and during his whole hospital stay. Response to treatment was mainly evaluated clinically, by resolution of low urinary tract symptoms.

Five months later, the patient was still LUTS-free; an abdomen and pelvic CT scan was performed, showing a significant decrease in the left ureter and bladder wall thickening and surrounding fat streaking (Figure 2).

## Background

To our knowledge, this is the first case report that identifies *T. pyogenes*/*A. pyogenes* as the cause of encrusted pyelitis in humans. It was shown to be the relevant pathogen since the patient failed many empiric antibiotic regimens but fully recovered only after appropriate antibiotic therapy with teicoplanin, and no other pathogen was isolated from the patient’s pelvis when culture was performed over regular and specific media.

Encrusted pyelitis was initially described in 1992 [2]. It is a rare chronic infectious and inflammatory condition of the renal pelvis characterized by calcific deposits or encrustation of the urothelium typically by phosphate and ammonium-magnesium salts [3,4]. It is a threatening disorder that can destroy the renal graft of transplanted patients but it can also affect native kidneys [5]. It has been described in immunocompromised or debilitated patients who have undergone urologic procedures to treat a variety of inflammatory or neoplastic conditions and patients treated with long-term broad-spectrum antibiotics. Reported cases are scant, even among renal transplant recipients who are at increased risk. Diagnosed patients are rare, with a reported incidence of 0.2% [6]. Alkaline-encrusted pyelitis is an infectious disease characterized by encrustations in the wall of the upper urinary tract, surrounded by severe inflammation [2]. For this reason, most consider it a healthcare-associated disease [7]. It is well-known in adults but rarely identified in children despite many case reports [8].

The disease is caused by an urea-splitting bacteria, most often *Corynebacterium urealyticum*, a Gram-positive, slow-growing microorganism that is multiresistant against antibiotics [2]. It has also caused one case of encrusted prostatitis [9], as well as severe fatal septic shock in immunocompetent patients [10,11]. It has even been responsible for nephrolithiasis associated with *C. urealyticum* urinary tract infection (UTI) in a dog [12]

Missing the diagnosis is not unusual since this germ has a lag phase higher than the regular conservation of urine culture (more than 48 h) and needs special culture media to grow in; therefore, it must be specified for the microbiologist. There is often a delay between the onset of symptoms and the diagnosis, which is usually more than 1 month duration in all cases [8].

The prevalence of infection by *C. urealyticum* and obstructive uropathy is increasing [13]. Clinical manifestations of encrusted pyelitis are nonspecific, including gross hematuria and pyuria, eventually with elimination of pus, urinary debris and stones [14]. Main complications of encrusted pyelitis include obstructive uropathy associated with end-stage renal failure, renal abscesses, ureteral stenosis and renal graft nephrectomy [15].

Imaging is essential for diagnosis. Thoumas *et al.* reported that the abdomen/pelvis CT scan without contrast is the baseline examination [16]. Calcifications covering the urothelium encrusting the wall of renal calyces, the renal pelvis, the ureter and the bladder must be distinguished from staghorn calculi [15,16]. CT scan is the modality of choice for the diagnosis and follow-up of the calcifications after treatment [17].

*Corynebacterium* species are always sensitive to vancomycin and teicoplanin [18]. Some authors recommend conservative management with antibiotics and oral dissolution of the plaques using urine acidifying agents such as ammonium chloride in capsule [19]. Percutaneous nephrostomy tubes allowing irrigation with Thomas’ acid solution are possible, the outflow being ensured by ureteric catheters [20]. Long-term follow-up is also necessary [21].

*Arcanobacterium pyogenes* aka *T. pyogenes* is a Gram-positive, pleomorphic bacillus, a commensal normal inhabitant of the mucous membranes of domestic animals, such as cattle, sheep, swine and goats and an opportunistic pathogen of economically important livestock, causing diseases as diverse as mastitis, liver abscesses and pneumonia [22,23]. It has not been isolated as part of the normal human flora [22] and is rarely a cause of infection in humans, with infections mostly related to living in rural areas and contact with animals [24]. There are three published reports of human *A. pyogenes* endocarditis in the literature [25], a case of sepsis in a farmer in Brazil [23] and three cases of wound infections associated with *A. pyogenes*, reported for the first time in India in patients with diabetes and a past history of Hansen’s disease, residing in a rural area in close contact with animals [26]. *A. pyogenes* is usually susceptible to benzyl penicillin, ampicillin, gentamicin and macrolides and resistant to cotrimoxazole, streptomycin and tetracyclines [27].

## Discussion

In the presented case, the persistence of severe LUTS despite prolonged previous antibiotic regimens raised suspicion of the likelihood of a multiresistant pathogen. In addition, CT scan findings of renal cortical calcifications and debris found on cystoscopy/ureteroscopy pushed the multidisciplinary team to further the investigations to isolate an uncommon pathogen incriminated in the sustained and recurrent UTIs. Although some findings were against encrusted pyelitis such as the urine pH of 6.5 (nonalkaline), the presence of debris in the patient’s pelvis and bladder in addition to cortical renal calcifications strongly pointed to the diagnosis, especially after the patient only improved after a teicoplanin regimen having failed many previous antibiotic therapies. Furthermore, it has to be kept in mind that the patient previously underwent an ureterovescial junction reimplantation, making it an additional risk factor of contracting encrusted pyelitits. The *Arcanobacterium* species identified in the patient’s culture was sensitive to penicillin, rifampicin, gentamycin, clindamycin vancomycin and resistant to sulfamethoxazole-trimethoprim, which is consistent with the known microbiology features of these bacteria. Pathogen sensitivity was tested through a susceptibility test performed *in vitro*, in a microbiology laboratory setting. Teicoplanin was the first and only antibiotic that was used to treat this patient.

## Conclusion & future perspective

In conclusion, the current report highlights the importance for urologists and infectious disease specialists to be alert to the possible diagnosis of encrusted pyelitis. In fact, despite encrusted pyelitis being a rare UTI, physicians should evoke this entity in the context of a UTI that is resistant to common treatment regimens or when no bacteria are found in cultures. It also strongly suggests that *A. pyogenes* aka *T. pyogenes* can be pathogenic to humans and should be considered by microbiologists for diagnostic and therapeutic purposes whenever a UTI with negative culture and atypical symptoms is present.

**Executive summary**

**Case presentation**

- A 69-year-old male presented with recurring lower urinary tract symptoms despite a complete course of antibiotic for prostatitis.
- Right open nephrolithotomy, left ureterovesical junction reimplantation, benign prostatic hyperplasia treated with tamsulosin are his past urologic history.
- Recurring macrohematuria and lower urinary tract symptoms with sterile urine cultures are his chief complaints.
- Abdomen and pelvis computed tomography (CT) scan showing multiscarred left kidney, renal pelvis calcification and thickening of his urothelial bed are suggestive of atypical infection or tumor.
- Renal pelvis cultures on special media showed *Trueperella pyogenes* growth.
- Intravenous Teicoplanin for 3 weeks resulted in resolution of his clinical symptoms and radiological features.

**Background**

- This is the first case report identifying *T. pyogenes* as the cause of encrusted pyelitis in humans.
- Encrusted pyelitis is usually caused by corynebacterial infection.
- Encrusted pyelitis results in alkaline urine pH, calcific deposits of the by phosphate and ammonium–magnesium salts.
- Diagnosis of encrusted pyelitis is usually missed and needs special media cultures.
- Abdomen/pelvis CT scan without contrast is the baseline examination.
- Corynebacterium species are always sensitive to vancomycin and teicoplanin.
- *Arcanobacterium pyogenes*, renamed *T. pyogenes*, is a Gram-positive, pleomorphic bacillus, a commensal normal inhabitant of the mucous membranes of domestic animals rarely pathogenic in humans.

**Discussion**

- Presence of debris in the patient’s pelvis and bladder in addition to cortical renal calcifications strongly pinpoint to the diagnosis of encrusted pyelitits (EP).
- Previous urologic surgeries are a risk factor for EP.
- Teicoplanin is the treatment of choice for EP.

**Conclusion & future perspective**

- It is important for urologists and infectious disease specialists to be alert to the possible diagnosis of encrusted pyelitis.
- EP should be evoked in the context of a urinary tract infection that is resistant to common treatment regimens or when no bacteria are found in cultures.
- *Arcanobacterium pyogenes* aka *T. pyogenes* can be pathogenic to humans and cause urinary tract infection.
